# Acyldepsipeptide Analogues: A Future Generation Antibiotics for Tuberculosis Treatment

**DOI:** 10.3390/pharmaceutics14091956

**Published:** 2022-09-15

**Authors:** Sinazo Z. Z. Cobongela, Maya M. Makatini, Phumlane S. Mdluli, Nicole R. S. Sibuyi

**Affiliations:** 1 Nanotechnology Innovation Centre (NIC), Health Platform, Advanced Materials Division, Mintek, Randburg 2194, South Africa; 2 Molecular Sciences Institute, School of Chemistry, University of the Witwatersrand, Johannesburg 2050, South Africa; 3 Department of Chemistry, Durban University of Technology, Durban 4000, South Africa; 4 Department of Science and Innovation (DSI)/Mintek Nanotechnology Innovation Centre (NIC), Biolabels Research Node, Department of Biotechnology, University of the Western Cape, Bellville 7535, South Africa

**Keywords:** acyldepsipeptides, antimicrobial peptides, caseinolytic protease, *Mycobacterium tuberculosis*, antimicrobial, tuberculosis, multi-drug resistance bacteria, antibiotics, proteolytic core, nanomaterials

## Abstract

Acyldepsipeptides (ADEPs) are a new class of emerging antimicrobial peptides (AMPs), which are currently explored for treatment of pathogenic infections, including tuberculosis (TB). These cyclic hydrophobic peptides have a unique bacterial target to the conventional anti-TB drugs, and present a therapeutic window to overcome *Mycobacterium Tuberculosis* (*M. tb*) drug resistance. ADEPs exerts their antibacterial activity on *M. tb* strains through activation of the protein homeostatic regulatory protease, the caseinolytic protease (ClpP1P2). ClpP1P2 is normally regulated and activated by the ClpP-ATPases to degrade misfolded and toxic peptides and/or short proteins. ADEPs bind and dysregulate all the homeostatic capabilities of ClpP1P2 while inducing non-selective proteolysis. The uncontrolled proteolysis leads to *M. tb* cell death within the host. ADEPs analogues that have been tested possess cytotoxicity and poor pharmacokinetic and pharmacodynamic properties. However, these can be improved by drug design techniques. Moreover, the use of nanomaterial in conjunction with ADEPs would yield effective synergistic effect. This new mode of action has potential to combat and eradicate the extensive multi-drug resistance (MDR) problem that is currently faced by the public health pertaining bacterial infections, especially TB.

## 1. Introduction

Tuberculosis (TB) has been terrorizing the world for millennia [1], yet the disease continue to thrive with none of the current therapy effective against the *Mycobacterium Tuberculosis* (*M. tb*) drug-resistant strains [2]. Efforts have been made to develop novel and effective anti-TB strategies with the goal to eradicate TB. Among others, natural products have played a significant role in drug discovery for decades and continue to make a significant contribution toward novel drug development [3]. Acyldepsipeptides (ADEPs) are emerging as potential anti-TB agents that are explored against *M. tb* strains, this is encouraged by the fact that they target different mechanism that are unique to the ones targeted by the conventional TB agents. As such, they might succeed where the TB agents are ineffective [4]. ADEPs were shown to have broad spectrum antimicrobial activity, including in drug-resistant *M. tb* strains [5]. However, none of the ADEPs are used clinically as TB treatment due to various challenges which include toxicity, solubility, metabolic instability, non-specificity, and membrane permeability. Safety and target specificity are the main aspects of drug development, and strategies have been put into place to improve on the pharmacokinetics of various drugs in order to improve patient compliance. To increase the bioavailability and therapeutic index of drugs, strategies such as chemical modification of the drugs, substitution of amino acids [6,7,8,9], and use of drug carriers were employed. Therefore, the same strategies can be used for modification and modulation of ADEPs to improve their targeting and overall pharmacokinetic properties. This review article presents an overview of TB and its impact on human health and economy; the conventional TB drugs, their mode of actions, and their limitations. ADEPs are presented herein as the upcoming and potential anti-TB agents; and lastly, the strategies that can improve their bioavailability and efficacy following strategies used for other drugs are also highlighted.

## 2. Tuberculosis

TB is a highly infectious disease and still one of the biggest killer, ranked among the top ten causes of death globally [10]. It is one of the oldest bacterial diseases as confirmed by the *M. tb* strain found in human remains dating back to at least 5000 BC [11]. TB has been a crisis to humans for millennia, and its emergence and causative agent was not understood until later in history [1]. At first it was suggested to be transmitted to human by *M. bovis* from cattle causing bovine TB and later believed that it was caused by inhalation of an infectious agent [12]. The TB causative agent was confirmed in 1882 by a German scientist Robert Koch who discovered that TB in humans was caused by a pathogenic *M. tb*. His postulates enabled the study of TB transmission and encouraged the essential discovery of acid-fast bacilli technique that is still widely used to date for the diagnosis of TB [13].

### 2.1. M. tb Epidemiology and Pathophysiology

TB was not considered an epidemic before the 17th century because of less infected population. However, due to urbanization in the early 17th century, the rate of human to human transmission of this airborne pathogen increased resulting in TB being declared an epidemic [14]. The mechanism of *M. tb* transmission in humans was detailed in 1930. Wells and colleagues made a conclusion that this bacterium is expelled from a person with an active pulmonary TB when coughing as 1-micron sized droplet nuclei that gets suspended in air. These droplet nuclei remain in the air and be inhaled by the next person [15]. Once inhaled into the lungs (alveolar) of the susceptible person, it has a potential to grow, causing new infection [16]. The infection can develop from inhalation of a single tubercle bacilli. These findings led to TB being declared as an airborne transmitted disease [17,18].

The progression of the infection varies in part due to the immune system of the host. There are four possible outcomes post inhalation of the tubercle bacillus. Developing the infection being the worst outcome and this usually occur to individuals with pre-existing immune compromising conditions such as human immune deficiency virus (HIV) [19,20]. Otherwise, the immune system has an ability to completely eradicate the bacterium before it launches an infection. In cases where the bacterium is not successfully eradicated, it can exist in a dormant stage known as latent TB infection [21]. This stage is asymptomatic and infection cannot be transmitted. However, at any given time ranging from immediate to decades, approximately 10% of latent TB-infected individuals can progress to active TB triggered by the deterioration of their immune system. Sometimes, latent TB can be activated when the *M. tb* escapes from the lungs to manifest in extrapulmonary sites such as the central nervous system, lymph node, bones, etc., [22].

Alternately, the bacterium can either come into contact with the resident macrophages or be ingested by the alveolar epithelial type II pneumocytes which lines the alveoli in the lungs. Pneumocytes are found in abundance than the macrophages in the lungs, and it is where the infection manifests [23]. Phagocytosis is activated by the bacteria encountering the macrophage mannose receptor [24]. This process triggers a glycoprotein (surfactant protein A) found in the surface of the alveolar to upregulate mannose receptor activity to promote phagocytosis of the bacteria [25]. Inside the macrophage, the *M. tb* is contained in a phagosome, an endocytic vacuole. Under normal phagosomal maturity cycle, the phagosome fuses with lysosome which introduces a hostile environment for the bacteria to survive. These include acidic pH, reactive oxygen intermediates, lysosomal enzymes, and some toxic peptides, with reactive nitrogen intermediates acting as the major elements in the antimicrobial activity of the macrophages [26]. With little knowledge on how and when in the stage of infection, polyclonal antibodies against *M. tb* bind and neutralize the active *M. tb*. The opsonized cells are also engulfed by the macrophages. Concurrently, the production of chemokines by infected macrophages recruits other immune cells such as monocytes, lymphocytes, and neutrophils. These help in formation of granuloma which increases cellular immunity to kill the bacilli-loaded macrophages to contain the spread of *M. tb*. However, the mycobacterium can survive the granuloma stage and continue multiplying and spreading in the alveolar or remain dormant for decades resulting in latent TB [27,28].

TB prevails in Africa as it is the poorest continent, facing most of the socio-economic problems such as unemployment, access to land, poor healthcare systems, etc. In the poor socio-economic class, people are obliged to reside in densely populated areas resulting in intense exposure and increased transmission rate. While TB is prevalent among the poor, it however has an effect across geographic boarders and social strata. Economically active age group, between the ages of 14 and 54 years old, accounts for three quarters of all TB cases with a death rate of 17%. This leads to loss and/or decreased income in affected households [29].

The World Health Organization (WHO) declared TB as a global public health emergency due to the epidemic proportions in some parts of the world and the rapid rise of MDR strains of *M. tb*. Before 2019, TB has previously been counted amongst the top 10 causes of death worldwide and a leading cause of death than HIV/AIDS. It was estimated that in 2020 TB caused about 1.3 million deaths, majority were HIV-negative cases and around 214,000 were HIV-positive people [30]. Even though there was a decline in TB diagnosis in 2020, it was estimated that about 10 million people developed TB. Out of the 10 million, 5.6 of those were men, 3.3 million were women, and about 1.1 million were children. The severity of this epidemic varies among countries; however, Africa and South-East Asia are still counted amongst the most prevalent continents [30].

In addition, 132,222 and 25,681 cases of rifampicin-resistant TB (RR-TB) and pre-XDR-TB global cases, respectively, for 2019 were reported by the WHO [30]. This shows an exponential growth of RR-TB and an alarming public health crisis as rifampicin is the most effective first-line TB drug. It was estimated that 82% of the RR-TB cases have MDR-TB. About 23% of the world’s population (1.7 billion) are estimated to have latent TB and are at a greater risk of developing the active TB disease later in their lives. The diagnosis and treatment of TB-infected people have helped to effectively avert millions of TB-related deaths.

### 2.2. Treatment of TB

The discovery of antibiotics is one of the most successful therapies to ever happen in the history of medicine. Antibiotics are antimicrobial agents produced by microorganisms in response to infections to inhibit growth or induce cell death upon interaction with microbial cells. Most TB antibiotics target the cell wall of the *M. tb* which is made up of peptidoglycan, polysaccharides, arabinogalactan, and mycolic acids [31]. To this day, the majority of the antibiotics currently used are mainly secondary metabolites produced by a number of microorganisms isolated from the soil. Antimicrobial agents are classified and named according to their mode of action by which they kill microorganisms [32]. Aminoglycosides, macrolides, etc., are known to inhibit the biosynthesis of proteins that are essential for bacterial cell homeostasis, thus causing cell death. Other antibiotics focus on disruption of the bacterial cell membranes, inhibition of folic acid metabolism, and inhibition of DNA replication and RNA synthesis. The conventional mode of action for most antibiotics, such as glycopeptides, is to target the bacterial cell wall biosynthesis. Penicillin is an example of β-lactam antibiotics that kill bacteria by specifically inhibiting the transpeptidase that catalyzes the final step in cell wall biosynthesis and the cross-linking of peptidoglycan [33].

It is widely known that penicillin was the first antibiotic discovered in 1928 by Sir Alexander Flemings [34], and it was only introduced into clinical practice in the 1930s [32,35]. Actually, in 1899, Emmerich and Low discovered pyocyanase [36], what we now call an antibiotic. Pyocyanase, extracted from *Pseudomonas aeruginosa*, was active against several pathogenic bacteria. It was the first antibiotic drug to be used clinically to treat various diseases. Unfortunately, pyocyanase was abandoned due to inconsistent treatment and its preparation was quite toxic to humans [37].

After a series of trials for compounds that were promising anti-mycobacterium by a soil microbiologist Selman Waksman, streptomycin was the very first successful TB drug introduced in 1947 [38]. Several active TB drugs were thereafter discovered and classified into two classes, the list of currently used anti-TB drugs and their mode of action is summarized in Table 1. The first-line drugs form a core treatment regimen, these include isoniazid, rifampicin, ethambutol, and pyrazinamide. Isoniazid is a prodrug which is activated by catalase peroxidase. Isoniazid inhibits an important enzyme (enoyl-acyl carrier protein reductase) component of fatty acid synthase II complex which is involved in the synthesis of mycolic acid and essential structure of the *M. tb* cell wall [39].

Second-line drugs are introduced to the regimen when dealing with resistant *M. tb* strains and these include but not limited to levofloxacin, moxifloxacin, bedaquiline, linezolid, and delamanid. In a case of XDR-TB, patients are given a combination of some of the first-line and second-line TB drugs with an addition of newly introduced drugs such as bedaquiline and delamanid [40,41,42].

**Table 1:** List of TB drugs and their mode of action.

TB Dugs			
Class	Drugs	Mode of Action	References
Thioamides	Ethionamide Prothionamide	Inhibit cell wall synthesis	[43,44]
Nitroimidazoles	Delamanid	Inhibit mycolic acid synthesis	[45]
Ethambutol		Inhibits cell wall synthesis	[46]
Cycloserine		Inhibits cell wall synthesis	[47,48]
Pyrazinamide		Exact target is unclear: Disrupts plasma membrane Disrupts energy metabolism	[49,50]
Diarylquinoline	Bedaquiline	Inhibits ATP synthesis	[51]
Aminoglycosides	Kanamycin Streptomycin	Inhibits protein synthesis	[52,53]
Cyclic peptides	Capreomycin	Inhibits protein synthesis	[54]
Fluoroquinolones	Moxifloxacin Levofloxacin	Inhibits DNA gyrase	[55,56]
Para-aminosalicyclic acid		Inhibit folate metabolism	[57,58]
**Recent TB Drugs**			
Nitroimidazoles	PA-24	Inhibits mycolic acid	[59]
SQ-109		Inhibits cell wall synthesis	[60]
Meropenem		Inhibits peptidoglycan synthesis	[61]
Benzothiazinones	PBTZ169 PBTZ043	Inhibits cells wall synthesis	[62,63,64]
Imidazopyridine amide		Inhibits cytochrome oxidase	[65,66]
Macrolides		Inhibits protein synthesis	[67,68]
Oxazolidinones	Linezolid Sutezolid	Inhibits protein synthesis	[69,70,71]

#### Bacterial Resistance as a Main Limitation for TB Drugs

The extensive use and prolonged courses of antibiotics to treat bacterial infections is causing an alarming increase in antibiotic resistance among Gram-negative and Gram-positive bacterial strains [72,73]. Most of the clinically relevant microorganisms have developed resistance toward almost all antibiotics known to man, including the first discovered antibiotic (penicillin). Among the β-lactam sensitive strains, a rapid increase in penicillin-resistance was reported against *Streptococcus pneumoniae* [74]. The mortality rate of penicillin-resistant *S. pneumoniae* infections is higher than that of penicillin-susceptible *S. pneumoniae* [75,76]. *M. tb* has been rapidly acquiring resistance toward most anti-TB drugs. This is mainly due to their hydrophobic thick cell wall composed of mycolic acid and other hydrophobic lipids such as phthiocerol dimycocerosates [2]. The cell wall components lead to difficulty in permeation of antibiotics to the bacterial cytoplasm [77]. The ability of the *M. tb* to be in a dormant state also increases their resistance to antibiotic treatment in their active state [78]. Hydrophilic TB antibiotics such as β-lactam use porin channels to penetrate *M. tb* cell wall [79]. However, *M. tb* modifies these porin channels to resist hydrophilic antibiotic uptake [80]. Additionally, *M. tb* possess efflux pump that help in expelling drugs that managed to infiltrate the bacterium [81]. *M. tb* also expresses anti-mycobacterium modifying and/or degrading enzymes [82,83].

*M. tb* strains that are resistant to various TB treatment, for instance rifampicin, isoniazid, pyrazinamide, ethambutol, and streptomycin have been reported. Rifampicin acts by binding to the bacterial DNA-dependent RNA polymerase *rpoB*-encoded β-subunit thereby inhibiting transcription. Rifampicin-resistance is caused by the mutation of 11 out of 12 amino acids within the *rpoB* gene that surrounds the rifampicin binding pocket [84]. MDR-TB is caused by *M. tb* that is resistant to at least rifampicin and isoniazid [42]. In the past two decades there has been an increase in the number of MDR cases [85,86,87] followed by XDR *M. tb* that is resistant to at least four of the core anti-TB drugs, mainly isoniazid, rifampicin, fluoroquinolone drugs, and at least one of the second-line injectables [88]. Recently, there has been *M. tb* strains that are resistant to all commercially available anti-TB drugs [89,90]. In low income countries, 8 out of 19 patients with XDR had *M. tb* that was resistant to all the anti-TB drugs leaving patients with no effective treatment [91].

Over 20 novel antibiotic classes were produced between 1930 and 1962 [92] and very few novel antibiotics have been marketed since then [93,94,95]. Despite the numerous numbers of novel antibiotics and their analogues, bacterial resistance is still a major clinical problem throughout the world. The emerging resistance of most pathogens to currently available antibiotics is raising an urgent need for new and effective antibiotic drugs. Exploring non-pharmaceutical compounds might give a wide range of chemical compounds utilized as effective antimicrobials. Over the years, peptides have been promising therapeutic agents for various diseases such as cancer [96], cardiovascular [97], diabetes [98], and others. There is an escalating attention toward synthetic analogues of natural antimicrobial peptides (AMPs) as potential and effective antimicrobial agents with novel mechanisms to combat the bacterial resistance problem [99,100].

## 3. AMPs as Future Antibiotics

AMPs, are host defense peptides that are endogenously produced by organisms for protection against pathogens [101]. AMPs are generally cationic with less than 50 amino acid residues [102]. They are part of innate immune response produced by eukaryotes and prokaryotes [103]. Cationic peptides exhibit diverse antimicrobial activities by enhancing bacterial agglutination, metal ion chelators, peroxidase activity, proteolytic inhibitors and impair cell wall activity [104]. These peptides have been proven to be active in vivo [105,106], effective against broad spectrum microbes [107,108,109] with reduced bacterial resistance [110] and side effects [111]. The antimicrobial activity of various AMPs have been reported against Gram-positive bacteria [9,112], Gram-negative bacteria [113,114], and fungi [115]. In addition, synthetic analogues of these peptides have demonstrated similar or enhanced activities compared to the natural AMPs. The synthetic peptides also have low minimum inhibitory concentration (MIC) [107], neutralizes the outer membrane lipopolysaccharides of Gram-negative bacteria [116], promote wound healing [117], and show synergistic activity with conventional antibiotics [118].

One of the first AMPs, named gramicidins, were isolated from *Bacillus brevis* in 1939 [119]. Gramicidins exhibited a bactericidal effect against a wide range of Gram-positive bacteria both in vitro and in vivo, and later was successfully used to treat bacteria-infected wounds in guinea-pigs skin [120]. As of August 2022, 3425 AMPs have been recorded in the Antimicrobial Peptide Database (APD3) isolated from microorganisms, plants, and animals as well as synthetic analogues. Examples include, magainins that were isolated from the skin of an African clawed frog (*Xenopus laevis*) [121] and purothionin from a common wheat plant *Triticumaestivum* [122]. The concentration of magainins that was found to be effective against pathogens was soluble in water and did not induce hemolytic effects. Magainins were proven to inhibit the growth of numerous bacterial species such as *Escherichia coli*, *Staphylococcus epidermidis* etc., and a known pathogenic fungi, *Candida albicans* [121,123]. Purothionin was found to be active against some pathogenic bacteria such as *Pseudomonas solanacearum* and *Xanthomonas campestris*, and fungi such as *Corynebacterium michiganense* [124]. These findings showed a promising future for AMPs as lead drug candidates against MDR microorganisms and possibly for effective TB treatment [7].

### AMP Mode of Action

**Figure 1:**
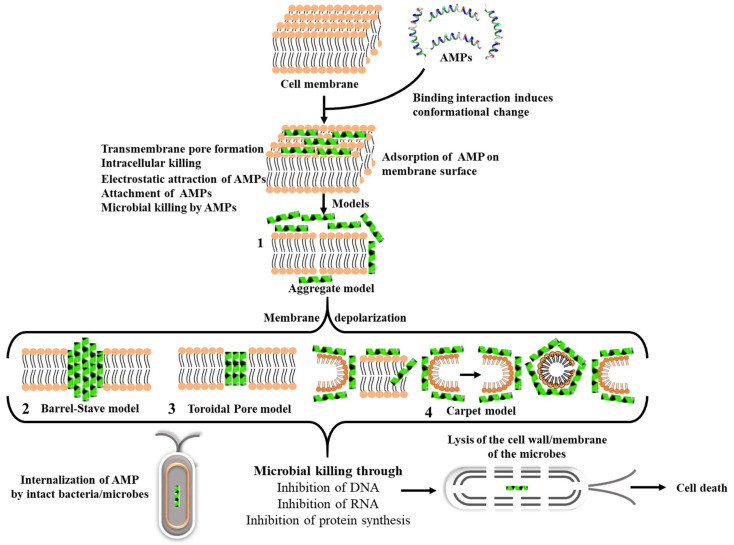
Antibacterial mechanism of AMPS. AMPs interact with bacteria and induce cell death following either aggregation (1), Barrel–Stave model (2), Toroidal Pore model (3) or Carpet model (4). Reprinted with permission from [127] published by MDPI, 2021.

When AMPs are introduced to living microorganisms, they trigger anatomical/functional changes at cellular and molecular levels. So far, disruption of membrane integrity and inhibition of intracellular activity have been identified as the two main mechanisms of action for AMPs [102]. In order to achieve this, AMPs must gain access to the cell by penetrating or interacting with the cell wall and/or cell membrane. Most of the AMPs have a cationic net charge due to their high content of the positively charged amino acid residues such as arginine, lysine, and histidine [125]. Due to the presence of lipopolysaccharides or teichoic acid, prokaryotes have a negative charge on their outer layer. In Gram-negative and Gram-positive bacteria, the cationic charge facilitates the accumulation of the AMP on the negatively charged surface of their outer membrane and cell wall, respectively [126]. The amphiphilic interaction between the AMPs and phospholipid bilayer of the bacterial cell wall/membrane is favorable in prokaryotes, this interaction encourages transmembrane pores which in turn disrupts the phospholipid bilayer and thus causing rapid cell lysis and eventually cell death [125]. Bacterial death by AMPs occur through similar mode of actions to those of antibiotics, by inhibition of DNA, RNA, and protein synthesis. AMP internalization can occur following one of the four mechanisms shown in Figure 1, i.e., aggregation, Barrel-Stave, Toroidal or Carpet models. All of these mechanisms are thoroughly explained elsewhere [127,128].

It is vital that AMPs gain access to the intracellular targets through the cell wall and/or cell membrane. The cell wall of Gram-positive bacteria embodies a porous (40–80 nm) thick mash where AMPs can pass through to the cell cytoplasm [129]. Alternatively, the cationic AMPs can also manipulate the charge exchange mechanism for transmembrane on Gram-negative bacteria where it competes with the Ca^2+^ and Mg^2+^ bound to lipopolysaccharide of the outer membrane [6]. Proline-rich peptides are one of the AMPs that target the intracellular processes to inhibit bacterial growth [8]. Once inside the cell, the AMPs may inhibit vital biological processes such as enzyme activity, nucleic acid synthesis, and protein folding or synthesis [126,130,131].

One of the examples of the AMPs that uses these properties to translocate to the cytoplasm is indocilin [132], investigations showed that it inhibits DNA synthesis thus inhibiting bacterial growth [133]. There are some AMPs that bind to both RNA and DNA such as buforin II causing rapid cell death [134]. PR-39 is a proline-arginine-rich peptide that was found to inhibit the growth of MDR *M. tb* by binding to DNA and inhibiting intracellular protein synthesis [135,136]. Though there are a number of intracellular AMP targets, bacterial proteases are one of the underexploited for new AMPs. There is a number of bacterial proteases such as ATP-dependent zinc metalloprotease complex such as caseinolytic protease (ClpP) complex that can be targeted as therapeutic targets [137].

## 4. ClpP as a Putative Bacterial Therapeutic Target

**Figure 2:**
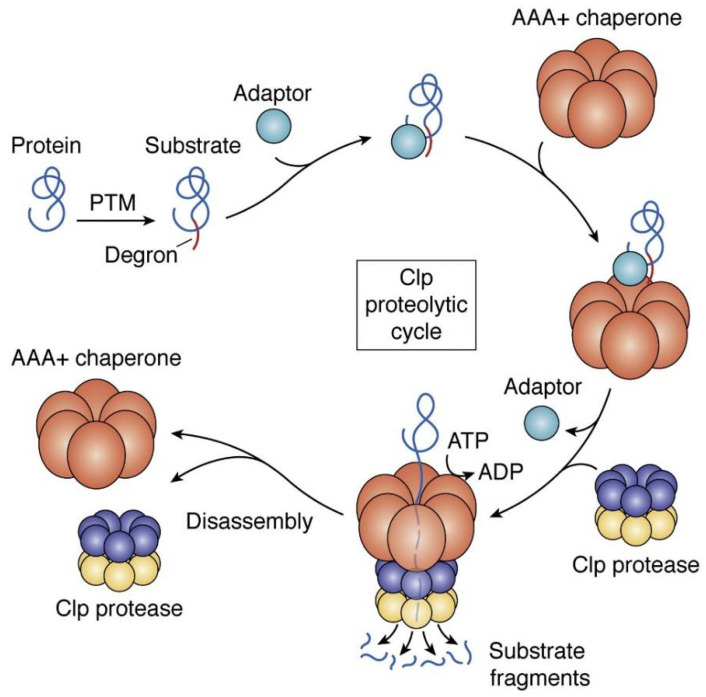
Schematic representation of the mechanism involved in regulated degradation of proteins by the ClpP complex. ClpP proteolytic activity is modulated by a series of events triggered by binding of misfolded/aggregated proteins to AAA+ chaperones. Reprinted with permission from [147] published by Elsevier, 2021.

ClpP is a serine peptidase that plays a crucial role in general protein quality control by degrading misfolded or aggregated proteins [138]. The ClpP belongs to a family of AAA+ ATPases and it is important for protein turnover and homeostasis in order to maintain vital cellular functions particularly under stress conditions. This protease is ATP dependent and referred to as proteolytic core [139,140]. It is made up of fourteen ClpP protomers that form a tetradecameric barrel-shape with two heptameric rings [141]. The catalytic residues are embedded within the barrel with seven hydrophobic pockets for interaction with the Clp-ATPase [142]. Clp-ATPase supports the refolding of proteins independent of ClpP, failure to achieve this, Clp-ATPase directs the misfolded or aggregated proteins to the proteolytic core where it is unfolded and degraded [143]. The misfolded proteins have a degron tag following the post-translation modifications [144]. The degron tag then binds to the adaptor protein which activates AAA+ ATPases to bind to the inactive ClpP [145]. Figure 2 illustrates the Clp proteolytic degradation cycle of the misfolded or aggregated protein. ClpP conformation plays a huge role in its catalytic activity, such that the compressed conformation is inactive with the increase in distance between the active site side chains of Ser 98, His 123, and Asp 172 while the extended conformation has these side chains at close proximity to allow interaction, thus it is active [141,146].

The role and importance of ClpP on the bacterial cell viability can then be explored as a target for antibacterial abilities. Currently, there are three mechanisms to deregulate ClpP i.e., disruption of the AAA+ ATPases coupling to the ClpP, the inhibition, and activation of the ClpP proteolysis chamber [137]. As mentioned above, ClpP is dependent on ATPases to select and unfold aggregated or misfolded proteins, disruption of this partnership deregulates the proteolytic activity of ClpP. Similarly, lassomycin and ecumicin have been proven to bind to the Clp-ATPase N-terminal domain and activate ATPase activity [148,149].

Lassomycin is a sixteen amino acid lasso-peptide produced by *Lentzea kentuckeyensis* [150]. It binds to the N-terminal domain of ClpC1 one of Clp-ATPase causing functional abnormalities in the ClpP [151,152] by increasing ATP hydrolysis. In theory, increase in ATP hydrolysis by ClpC1 is associated with increase in protein degradation. Lassomycin prevents translocation of proteins which further inhibits the proteolysis by ClpP [151]. Lassomycin binds to the N-terminus domain of ClpC1, a highly acidic region encouraged by its four positively charged amino acids [153,154]. The binding site on the ClpC1 has arginine21 and proline79; however, the most critical binding site was glutamine17 which forms a hydrogen bonding with lassomycin. The bactericidal activity of lassomycin is assumed to be its ability to increase ATP hydrolysis by ClpC1 thus leading to ATP depletion in the cell and/or excessive protein unfolding [151].

Ecumicin is a cyclic tridecapeptide extracted from *Nonomuraea* sp., like lassomycin, it binds to the N-terminal domain (glycine1-tyrosine145) of ClpC1 [150,151]. It also increases ATP hydrolysis by ClpC1 by blocking substrate/protein recognition by ClpC1 therefore inhibiting proteolysis [155]. It is assumed that there are hydrophobic interactions between ecumicin and ClpC1, furthermore, ecumicin forms two hydrogen bonds with the N-terminal domain of ClpC1 [151,155]. It was suggested that arginine83 of ClpC1 is important for interaction with ecumicin [155]. Ecumicin was found to act in a similar manner to MecA, an adaptor protein that enhances the hexamerization of ClpC, thus increases the ATPase activity [149,155].

Other molecules such as β-lactone, focus on inhibiting the proteolytic core of the ClpP, where they covalently bind to Serine98 thus irreversibly inhibiting the proteolytic activity [156]. Interestingly, this mechanism was also shown to inhibit the growth of *M. tb*, suggesting that the ClpP could be an important target for the TB therapeutic intervention. For instance, cyclomarine A, a natural cyclic heptapeptide produced by *Streptomyces* sp., was shown to have high affinity and specificity for ClpC1 ATPase of *M. tb* by binding to the N-terminal domain of ClpC1 [149,151]. Most importantly, activation of the ClpP protease is distinctly an option that is exploited for development of therapeutic AMPs for various infectious diseases, including TB. Exploring this option of activation rather than inhibition of the ClpP may be effective against dormant or drug resistant bacteria. Since ADEPs have been reported to be using this antimicrobial mechanism, they can be explored as TB therapy [137].

### 4.1. ADEPs Competes with Clp-ATPases to Deactivate ClpP

**Figure 3:**
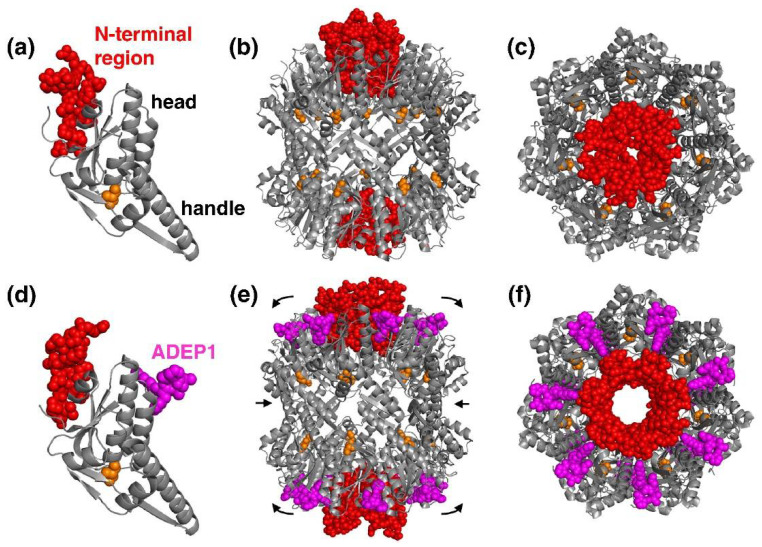
X-ray structure of inactive and active ClpP. (**a**) Inactive ClpP N-terminal domain (red). N-terminal residues from 1-18 are highlighted in red and the nucleophile active site is highlighted in orange. (**b**) Equatorial view of the inactive ClpP. (**c**) Axial view of the inactive ClpP. (**d**) ADEP1 (purple) bound active ClpP N-terminal domain. (**e**) Equatorial view of the ADEP1 activated ClpP. (**f**) Axial view of the ADEP1 activated ClpP. Reprinted with permission from [162], published by Elsevier, 2013.

ADEPs, also known as cyclic ADEPs, are a class of AMPs that bind and deregulates bacterial ClpP [4]. They are naturally produced by *Strepomyces hawaiiensis* [157] and are most active against Gram-positive bacteria [137]. ADEPs bind to the cavities formed by two of ClpP monomers. ClpP/ADEP complex adopts a proteolytic active conformation in the absence of Clp-ATPase [4,158]. ADEPs mimic the Clp-ATPases activity and competitively binds to the hydrophobic pockets. This event inhibits interaction between ClpP and Clp-ATPases, and therefore eliminates all natural functions of the Clp-protease that require Clp-ATPase-mediated degradation [159,160,161]. Figure 3 displays the X-ray structure of unbound ClpP N-terminal region and ADEP ClpP complex. Upon binding of the ADEP or Clp-ATPase, ClpP let loose of its N-terminal of the β-loop which on an intact state prevents ClpP from randomly going through degradation [159,160]. ADEPs or Clp-ATPase increases the proteolytic activity of ClpP thus degrading proteins (both essential and nonessential) around the target cells leading to cell death.

The catalytic core of the ClpP has three amino acid residues, Serine98, Histidine123, and Aspartic acid172, that form a catalytic triad [163]. On the active conformation of ClpP, Serine98 undergoes a nucleophilic attack on the electron deficient carbonyl group of the peptide bond. The imidazole ring on Histidine123 abstracts a proton from the serine hydroxyl group and the resultant positively charged histidine imidazole ring is stabilized by the carboxyl function of the aspartic acid172. The aspartate acyl-ester undergoes hydrolysis regenerating the serine for the next catalytic cycle [141].

The aspartic acid and threonine side chains create a polar environment whereas the aliphatic side chain and benzene ring of the phenylalanine are buried deep into the hydrophobic pocket of ClpP [159]. The aliphatic side chain and N-acylphenylalanine moiety closely resembles the isoleucine/leucine-glycine-phenylalanine structure of one of Clp-ATPase (ClpX) hence this is a minimum structural requirement for ADEP activity [164]. Moreover, Tyrosine62 residue form two hydrogen bonds with N-group of phenylalanine e and a carboxylic group of alanine thus increasing rigidity between ADEP and ClpP [159]. The ADEP1 Factor A (A54556) is one of the ADEPs to be discovered [4], and has been reported to have antibacterial activity against Gram-positive bacteria [5] and Gram-negative bacteria [165]. ADEP1 was also reported to be bactericidal against a number of antibiotic-resistant Gram-positive bacteria such as penicillin-resistant *S. pneumoniae*, vancomycin-resistant *Enterococcus faecium*, and methicillin-resistant *Staphylococcus aureus* [5].

### 4.2. ADEP1 Analogues

**Figure 4:**
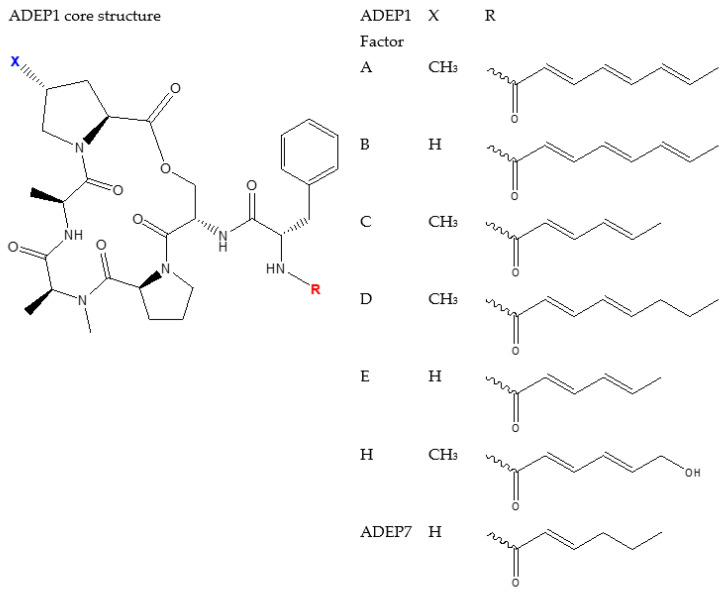
Structures of ADEP analogues modified from a core structure of ADEP1.

**Figure 5:**
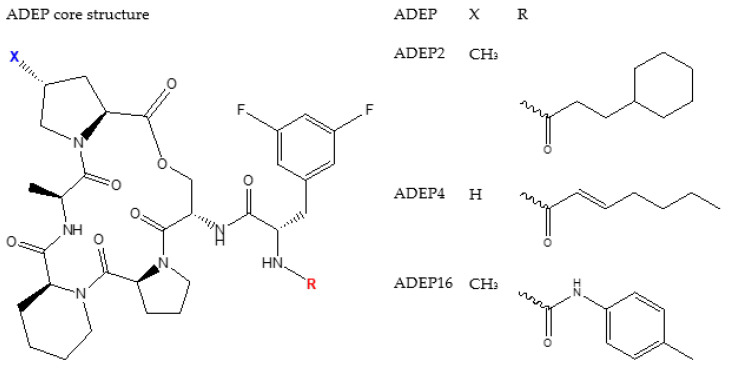
Structures of ADEP analogues modified from a core structure with pipecolic acid.

**Figure 6:**
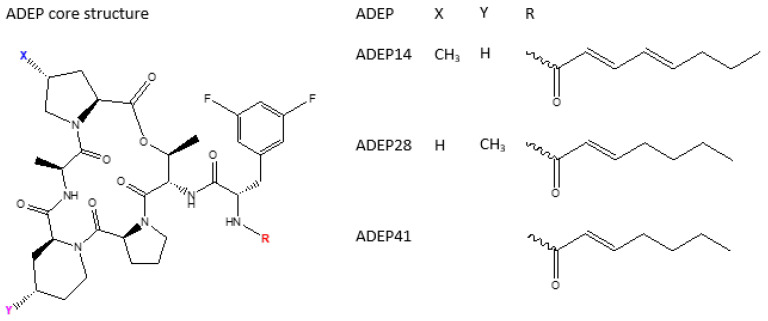
Structures of ADEP analogues modified from a core structure with Ser replaced by Thr.

**Figure 7:**
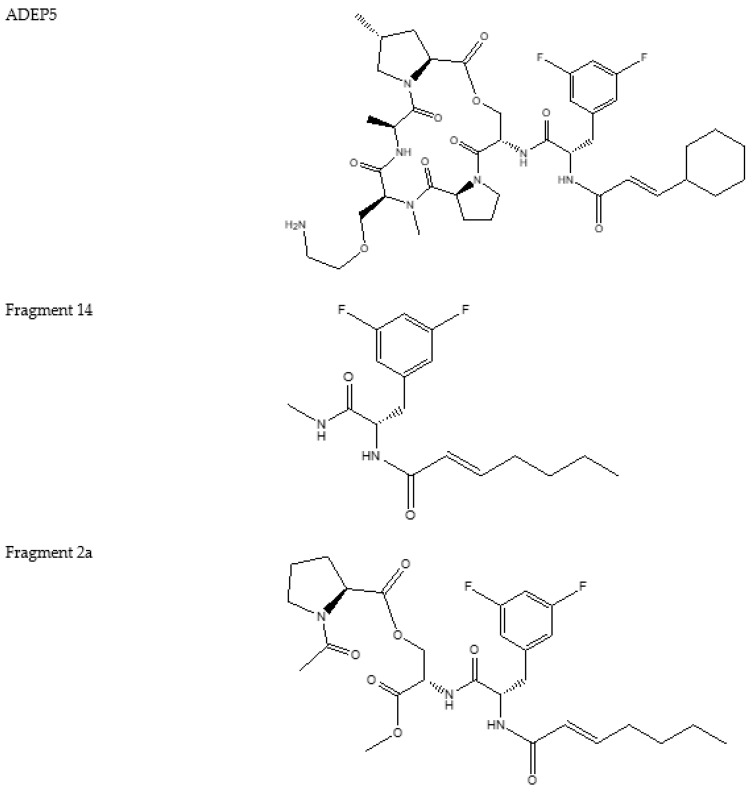
Structures of ADEP analogues modified from a core structure with 3-methoxypropylamine and ADEP fragments.

There is a number of modification that have been done on the original ADEPs to yield better pharmacokinetics and pharmacodynamics. Figure 4, Figure 5, Figure 6 and Figure 7 show some of the synthesized and tested ADEP analogue structures and fragments. ADEP1 Factor A (Figure 4) is cyclic peptide composed of four natural amino acids (proline, alanine, serine, and phenylalanine), two methylated amino acids (4-methyl proline and N-methylated alanine) and octa-2,4,6-trienoic acid. ADEP1 Factor A is active against various microorganisms including *Bacillus subtilis*, *S. pneumoniae*, *Streptococcus pyogenes*, *Enterococcus faecalis*, *E. faecium*, *S. aureus* [4,165], etc. The very first modification was the removal of the methyl group from proline (ADEP1 Factor B) which is active against *S. aureus*, *E. faecalis*, and *S. pneumoniae* [165,166]. The removal of the methyl proline in Factor B led to a decrease in antibacterial activity in *S. aureus* [165,166]. Other ADEP1 analogues have been used in different types of enoic acids (ADEP1 Factor D) to reduce the number of double bonds in octa-2,4,6-trienoic acid for thermal stability [165]. This analogue was active against a number of Gram-positive (Methicillin-resistant *S. Aureus*, vancomycin-resistant *enterococcus*, and penicillin-resistant *S. pneumoniae*) and Gram-negative pathogenic bacteria (*Neisseria meningitidis* and *Neisseria gonorrheae*) [5,167]. The use of fluorinated *bis*-fluorophenylalanine side chain in a place of phenylalanine has shown enhanced antibacterial activity of ADEP2, ADEP4, and ADEP5 on *S. aureus* [4]. Modifications have also aimed in rigidifying the macrolactone core. This was achieved by replacing the N-methyl alanine on the macrolactone core with pipecolic acid to yield ADEP2, ADEP4, ADEP14 as shown in Figure 5 [4,168,169], etc. The rigidified ADEP4 and its analogues still maintained the antimicrobial activity against *S. aureus*, *S. pneumoniae*, and *E. faecalis* [169]. ADEP14, ADEP28, and ADEP41 (Figure 6) were achieved by replacing serine with threonine. Fragments of the ADEPs (Figure 7) have also been tested for the ClpP activation and antimicrobial activity. Two fragments still maintained the antimicrobial activity against *B. subtilis* [170].

### 4.3. ADEPs Activity on M. tb ClpP1P2

ADEPs have particularly been of interest in *M. tb* targeting due to its distinct mode of action on ClpP [4]. The *M. tb* ClpP not only maintains intracellular protein homeostasis, it also contributes to the mycobacterium virulence and helps with the dormancy of the *M. tb* within the host [171,172,173]. Unlike other bacteria with a single gene for ClpP, *M. tb* has two genes encoding for ClpP1 and ClpP2 proteolytic subunits [174]. Like other ClpP, ClpP1 and ClpP2 heptamer rings are inactive until they associate to form a 300 kDa ClpP1P2 tetradecamer [152,171]. Upon binding together, both these rings influence each other’s conformation [152,175]. In vitro, ADEPs exclusively bind to the *M. tb* ClpP2 which in turn changes the whole ClpP1P2 tetradecamer conformation to open both heptamer axial pores thus activating the ClpP1P2 [175]. This phenomenon is naturally controlled by ATPases in response to increased concentration of substrates. In low concentrations of substrates, ATPase-bound ClpP1P2 remains inactive. However, ClpP1P2 activation by ADEPs is independent of the amount of substrate present. The anti-mycobacterium activity of ADEPs and the exact mechanism of how they bind thus causing bacterial growth inhibition through ClpP1P2 is poorly understood.

It has been previously reported that the anti-mycobacterium activity of ADEPs is through nonselective protein degradation by *M. tb* ClpP1P2, and/or allosterically binding to ClpP1P2 to prevent physiological activity of the ATPase [175]. Anti-mycobacterium activity of ADEPs is also through ClpP1P2 inability to degrade and eliminate toxic proteins [176]. The ADEPs and analogues bind to leucine-glycine-phenylalanine hydrophobic pockets on the ClpP2 which is the same binding site for the ATPases [177,178]. A list of ADEP analogues (ADEP2, ADEP3, ADEP4, IDR-10001, and IDR-10011) were found to be active against *M. tb* with an MIC range between 25 and 100 µg/mL [179]. Although the ADEP binding to the ClpP1P2 is similar to other bacterial ClpP complexes, the maximum effect of ADEPs on ClpP1P2 require activators such as dipeptide benzyloxycarbonyl-leucyl-leucine [176]. ADEPs were also shown to have improved activity with addition of efflux pump inhibitors which prevents export of intracellular ADEPs [81,179] and therefore, have potential to be effective where the current anti-TB drugs have failed.

### 4.4. Limitation of ADEPs

The problems mainly associated with novel therapeutic AMPs, including ADEPs are their poor pharmacokinetic properties such as susceptibility to enzyme degradation, short plasma half-life (leading to frequent invasive administration), lack of oral availability, difficulty in membrane permeability, lack of selectivity thus causing toxicity to normal cells [180,181]. These complications raise a need to improve the natural peptides in drug like molecules with less toxicity, high selectivity, good solubility, increased proteolytic stability, and membrane permeability. This in turn will increase drug bioavailability and consequently increasing their therapeutic activity. Therefore, modification and modulation of conformational dynamics of these AMPs and designing of similar systems that mimic the potential therapeutic peptides will be the first step toward improving the pharmacokinetic properties of the AMPs. There are several ways that can be employed to improve the pharmacokinetic properties of novel peptides. Peptide modification and use of drug carriers are some of the strategies that have been successful in enhancing the bioactivity of AMPs and their analogues [6,7,8,9].

#### 4.4.1. Potential Strategies to Improve on ADEP’s Pharmacokinetics and Pharmacodynamics

ADEPs are produced by natural sources such as plants and microorganisms, often in minute quantities and majority of them are less soluble, which presents a huge challenge for their application in therapeutics. Drug modifications has been successful in improving the membrane permeability, drug stability, and controlled delivery at the target site. Some of the strategies that can be employed to prevent ADEPs biodegradation or interaction with biomolecules include chemical modification of the drugs, substitution of amino acids [6,7,8,9], and use of drug delivery agents.

##### Chemical Modification

Chemical modification and amino acid substitution in lead drugs, no matter how small it is, have significant impact on their bioactivity; a lesson learnt from nature, where a slight change in a structure can completely alter its function, target, mode of action and improve drug selectivity [182]. Changing the drug structure conformation through cyclization is another way of increasing their metabolic stability compared to linear analogues. Cyclization can be formed through chemically stable bonds such as ether, disulphide, lactone etc., and the most common technique used in peptide chemistry [183].

##### Amino Acid Substitution

Protease stability is also vital in the development of peptide-based drugs, the incorporation of unnatural amino acids is one of the approaches used to increase their stability and bioavailability. There is quite a number of processes to achieve these unnatural amino acid analogues such as, substitution with either cationic [184], D-amino acids or N-methylated amino acids [185], can help improve on the metabolic stability and potency of peptide-based therapies. For instance, cationic peptides exhibit more bactericidal effects than their anionic counterparts. Therefore, replacing negatively charged amino acids (aspartic acid and glutamic acid) with positively charged ones (lysine and arginine) increases the positive net charge of the peptides. Moreover, lysine and/or arginine rich peptides easily interact with the negatively charged cell membrane and are used to shuttle biomolecules across the cell membrane [184]. The cationic peptides will therefore serve dual functions as cell-penetrating peptides and antimicrobial agents [186,187]. Additionally, substituting the natural L-amino acids with D-amino acids may be used to increase proteolytic stability of the peptide drugs. The L-amino are easily metabolized by the body and are susceptible to protease degradation. D-amino acids on the other hand, have similar activity to L- amino acids but are not susceptible to degradation, thus improving their bioavailability and drug activity [188,189]. N-methylation and fluorination of the amino acids also improve pharmacokinetic characteristics, activity, selectivity, and delivery of peptides [185]. N-methylation also improved metabolic stability and intestinal permeability of peptides that were highly active but with poor bioavailability [190]. Nature has also employed N-methylation of peptides on the ADEP1 to improve its biological functions and mode of survival by inhibiting enzymatic degradation [165]. Multiple N-methylation also increased selectivity of a cyclic hexapeptide integrin antagonist toward different integrin subtypes [185,191]. Attention has also been given to fluorination to modulate physicochemical properties of proteins, especially of hydrophobic amino acids (phenylalanine, isoleucine and others), to increase peptide stability and prevent proteolytic degradation [192].

##### Lipophilic Molecules

Lipophilic molecules were also used to improve and facilitate the cellular uptake of AMPs or ADEPs as their mode of action is targeted on intracellular proteins. Lipophilic molecules such as linoleic, oleic, and palmitic acids are often conjugated to the drugs to increase membrane permeability of peptide-based drugs. They are mostly used in pharmaceuticals to enhance uptake of chemicals [193]. The conjugation of lipids to drugs increases their lipophilicity which enhances their interaction with the cell membrane thus increasing cellular uptake. Amongst other advantages of lipid-drug conjugates, they also improve the oral bioavailability and decrease toxicity of the drug molecule [194].

##### Nano-Carriers

The urgent need for development of new drug delivery systems with improved properties to achieve desirable therapeutic efficacy is driven by the toxicity and side effects of drugs. Nanomaterials have a wide range of applications in various fields. In the pharmaceutical industry, they are receiving significant interest and are being investigated for drug formulation and delivery (nanomedicine) [195]. Nanomaterials are very small in size, usually ranging from 1 to 100 nm, and yet have a larger surface area that can be easily manipulated by conjugating compounds of interest [196]. Because these particles are too small, they easily diffuse through cell membrane pores and ion channels, and their target specificity can be further improved when the targeting ligands are attached to nanomaterials [197]. Nanomaterials as drug carriers can also help in increasing drug solubility therefore increasing bioavailability of drugs [198]. Additionally, they can reduce toxicity and side effects by increasing selectivity and can improve transfer across membranes including the blood–brain barrier [199] without the aid of targeting moieties. These conjugates also decrease enzyme susceptibility of unstable drugs at physiological conditions [200]. There are several organic and inorganic nanomaterials that are promising drug delivery agents. Inorganic or metal nanoparticles such as zinc, copper, and iron oxides nanoparticles have been explored for this application, mostly due to their antibacterial activity.

The nanomaterials have been shown to exhibit antimicrobial properties against a wide range of pathogens including drug-resistant strains. For example, copper oxide (CuO) nanoparticles were effective against methicillin-resistant *S. aureus* and *E. coli* [201,202]. When loaded with drugs, they can translocate their cargoes across the cell membrane. Iron oxide (FeO) nanoparticles loaded with doxorubicin could transport doxorubicin across cellular membranes without any targeting moiety and accumulate in the nucleus [203]. The nanoparticles increased the selectivity and bioavailability of the drugs to the target cells and at the same time reduce the side effects. Nanomaterials such as zinc oxide (ZnO) nanoparticles were also shown to possess anti-TB activities, and could be used as both antimicrobial as well as drug delivery agents. Interestingly, bimetallic NPs, in this case mixing ZnO with silver nanoparticles improved their biocompatibility and reduced the toxicity resulting from individual metal nanoparticles in *M. tb* [204]. Thus, NPs alone or in combination with targeting molecules such as antibodies or targeting ligands can help improve the potency and bioavailability of ADEPs.

## 5. Conclusions

ADEPs bind and dysregulate bacterial ClpP complex leading to uncontrolled proteolysis of proteins that are essential for bacterial survival. They have proven to be effective antimicrobial and/or bactericidal agents in a number of bacterial strains including the drug-resistant mycobacterium. Even though ADEPs are highly potent antimicrobial agents, they have poor pharmacokinetics and pharmacodynamics properties to be used as active pharmaceutical agents. Since their discovery about 15 years ago, much work has been carried out to modify these peptides to yield better results. Some modifications were successful, while others diminished their antimicrobial effect. There is still more to be done regarding these antimicrobial peptides. Especially the modification of ADEPs to increase the binding ability to the ClpP1P2 without the help of activators. This includes changing the cyclization bond and using enantiomers, lipophilic molecules, and drug carriers. Moreover, the strategic use of cationic amino acids would increase the interaction with the highly negative bacterial cell wall/cell membrane. This in turn would increase the solubility and cell penetrative ability of ADEPs. Another approach to overcome this tailback is the conjugation of ADEPs to nanomaterials. This approach will not only increase the drug delivery, but it will also increase drug availability and effectiveness of the ADEPs. Nanomaterials have been used as effective antimicrobials, thus can synergistically work with the cargoes toward eradicating TB.

## Data Availability

Not applicable.
